# How to assess severity in males with eating disorders? The DSM-5 severity index versus severity based on drive for thinness

**DOI:** 10.1192/j.eurpsy.2021.1864

**Published:** 2021-08-13

**Authors:** A. Dang, I. Krug, R. Granero, Z. Agüera, I. Sánchez, N. Riesco, S. Jimenez-Murcia, F. Fernandez-Aranda

**Affiliations:** 1 Melbourne School Of Psychological Science, University of Melbourne, Melbourne, Australia; 2 Department Of Psychobiology And Methodology, Universitat Autònoma de Barcelona - UAB, Barcelona, Spain; 3 Department Of Public Health, Mental Health and Maternal-Child Nursing, School of Nursing, University of Barcelona, Barcelona, Spain; 4 Ciber Fisiopatología Obesidad Y Nutrición (ciberobn), Instituto Salud Carlos III, Barcelona, Spain; 5 Department Of Psychiatry, University Hospital of Bellvitge-IDIBELL, Barcelona, Spain

**Keywords:** Males, DSM-5 severity indicators, anorexia nervosa, Bulimia Nervosa

## Abstract

**Introduction:**

The Diagnostic and Statistical Manual of Mental Disorders-5 (DSM-5) introduced severity indices for Eating Disorders (ED).

**Objectives:**

This study assessed in a male ED sample the DSM-5 severity indices for Anorexia Nervosa (AN), Bulimia Nervosa (BN) and Binge Eating Disorder (BED) and compared them to an alternative transdiagnostic drive for thinness (DT) severity category and a combined DSM-5/DT severity categorization

**Methods:**

178 males with EDs were classified using: a.) a DT categorisation based on the EDI-2 DT subscale; b.) the DSM-5 severity categories for AN, BN and BED and c.) a combination of the DT and the DSM-5 severity categorisation. These severity classifications were then compared based on psychopathology and personality.

**Results:**

For the DSM-5 severity indices, the “mild” category was most prevalent for AN and BN, and the “moderate to extreme” group for BED. For the EDI-2 DT severity classification, the “mild” category was overrepresented in all subtypes. For the combined DSM-5/DT categorization, the “mild combined” severity group was the most prevalent for AN, while for BN and BED the “severe/extreme” combined group was most prevalent. Clinically significant findings were strongest for the DT categorization followed by the combined DSM-5/DT approach. Almost non-significant findings were revealed for the DSM-5 severity categories for all ED subtypes. These findings were most pronounced for AN and BN and almost non-existent for BED.

**Conclusions:**

Our findings provide support for DT as an alternative transdiagnostic severity category for EDs in males that may be more meaningful than the DSM-5 severity indices for AN and BN, but not BED.

**Disclosure:**

No significant relationships.

